# Predicting global QoL after orthotopic neobladder or ileal conduit diversion: nomogram development

**DOI:** 10.3389/fonc.2023.1055140

**Published:** 2023-05-10

**Authors:** Salvatore Siracusano, Agustina Zaka, Emanuele Zaffuto, Antonio Benito Porcaro, Renzo Colombo, Renato Talamini, Federico Romantini, Francesco Montorsi, Cristina Lonardi

**Affiliations:** ^1^ Department of Urology, University of L’Aquila, L’Aquila, Italy; ^2^ Department of Urology, University of Verona, Verona, Italy; ^3^ Department of Urology, Vita e Salute San Raffaele University, Milan, Italy; ^4^ Epidemiologist Freelance Consultant in Epidemiological Studies and Clinical Trials, Aviano, Italy; ^5^ Department of Human Sciences, University of Verona, Verona, Italy

**Keywords:** bladder cancer, orthotopic neobladder, ileal conduit, nomogram, quality of life (QoL)

## Abstract

**Introduction:**

Quality of life (QoL) outcomes in patients undergoing radical cystectomy (RC) with orthotopic neobladder (ONB) or ileal conduit (IC) have been extensively investigated. However, a general lack of consensus on QoL’s predictive factors exists. The aim of the study was to develop a nomogram using preoperative parameters to predict global QoL outcome in patients with localized muscle-invasive bladder cancer (MIBC) undergoing RC with ONB or IC urinary diversion (UD).

**Methods:**

A cohort of 319 patients who underwent RC and ONB or IC were retrospectively enrolled. Multivariable linear regression analyses were used to predict the global QoL score of the European Organisation for Research and Treatment of Cancer Quality of Life Core Questionnaire (EORTC QLQ-C30), according to the patient characteristics and UD. A nomogram was developed and internally validated.

**Results:**

Patients’ data in the two study groups significantly differed with regard to comorbidity profiles (chronic cardiac failure, p < 0.001; chronic kidney disease, p < 0.01; hypertension, p < 0.03; diabetic disease, p = 0.02; chronic arthritis, p = 0.02). A multivariable model that included patient age at surgery, UD, chronic cardiac disease, and peripheral vascular disease represented the basis for the nomogram. The calibration plot of the prediction model showed a systematic overestimation of the predicted global QoL score over the observed scores, with a slight underestimation for observed global QoL scores between 57 and 72. After performing leave-one-out cross-validation, the root mean square error (RMSE) emerged as 24.0.

**Discussion/conclusion:**

A novel nomogram based completely on known preoperative factors was developed for patients with MIBC undergoing RC to predict a mid-term QoL outcome.

## 1 Introduction

Bladder cancer is the 10th leading cancer worldwide, and its incidence is steadily rising globally, especially in developed nations (1).

According to the European Association of Urology (EAU), it is well known that neoadjuvant chemotherapy followed by radical cystectomy (RC) with urinary diversion (UD) using an ileal conduit (IC) or an orthotopic neobladder (ONB) is the gold standard treatment for localized muscle-invasive bladder cancer (MIBC) and recurrent, high-risk non-muscle-invasive bladder cancer (NMIBC) (2). Nevertheless, this surgery is a morbid procedure that deeply affects daily social, sex, and working life (3, 4), with a general lack of consensus on predictive factors of quality of life (QoL) contrary to what happens for other types of surgical outcomes (5). For this reason, in the last decades, there was an exponentially growing interest in QoL investigations in cystectomized patients by comparison of continents with incontinent UD (6–9) but without expectations on the QoL in the short-term and mid-term; therefore, it is impossible to plan adequate counseling for the patient.

The aim of this study was to develop a multivariable model using preoperative parameters to predict the global QoL outcome measured with the validated Italian version of the European Organisation for Research and Treatment of Cancer Quality of Life Core Questionnaire (EORTC QLQ-C30) questionnaire in patients with MIBC undergoing RC with ONB or IC diversion in order to enhance the case management process.

## 2 Materials and methods

We analyzed retrospectively a multi-institutional database of 319 patients alive at the time of the study with a median follow-up of 48.5 months who had undergone RC and UD for urothelial (BC in five high-volume Italian urological centers between June 2007 and September 2013 as a part of a study approved and funded by the Italian Ministry of Scientific Research. All patients were older than 18 and were affected by MIBC or by recurrent, high-risk NMIBC as suggested by EAU Guidelines (2). They had all undergone RC performed by experienced surgeons as described by Skinner and Lieskovsky (10, 11) followed by UD by either IC or ONB. ONB were VIP nobladders (Vescica Ileale Padovana). All patients at the time of enrollment for surgery were without cognitive or cerebral impairment, without a history of psychiatric disorders or substance abuse, and were able to understand and fill out a QoL questionnaire during the follow-up visit. All patients enrolled in the study provided written informed consent.

### 2.1 QLQ-C30 questionnaire

QoL was measured postoperatively and during follow-up checkups at 1, 3, or 5 years after RC by using the EORTC QLQ-C30 questionnaire, which is a modular 30-item questionnaire developed and copyrighted by the EORTC and designed to assess the QoL of cancer patients participating in clinical trials (12, 13). The questionnaire is composed of nine multi-term scales, as follows: five functional scales (physical, role, cognitive, emotional, and social), three symptom scales (fatigue, pain, and nausea/vomiting), a global health and QoL scale, and items assessing the perceived financial burden of cancer and other symptoms frequently reported by cancer patients such as constipation, diarrhea, dyspnea, loss of appetite, and sleep disturbance (14). We used the validated Italian version of the EORTC QLQ-C30 (15). The majority of questions were assigned a score from 1 to 4 (1 = not at all, 2 = a little, 3 = quite a bit, and 4 = very much). Global health and QoL scales were assigned a score from 1 to 7. As suggested in the EORTC Manual scoring, we linearly transformed all variables to a 0–100 scale (16). Data were collected from each of the patients through an individual interview conducted in the outpatient clinic in the course of the follow-up visit.

### 2.2 Variable definition and statistical analyses

Patient characteristics included age at surgery, gender, and body mass index (BMI; kg/m^2^). Comorbidities were assessed using the Charlson comorbidity index (CCI), which assesses comorbidity level by taking into account both the number and severity of 19 pre-defined comorbid conditions. The endpoint of interest consisted of testing the ability of patient baseline characteristics and UD to independently predict the post-operative EORTC QLQ-C30 global QoL score. Frequency tables were used to analyze patients’ characteristics, according to UD. Chi-square and Mann–Whitney tests were used to evaluate associations between categorical and continuous variables, respectively. Multinomial linear regression analyses were used to predict the EORTC QLQ-C30 global QoL score, according to the patient characteristics and UD. Internal cross-validation was performed to test the validity of the model. Finally, a preliminary nomogram was developed to ease the calculation of the predicted global QoL score.

All statistical testing was two-sided with a level of significance set at p < 0.05. Analyses were performed using the R software environment for statistical computing and graphics (version 3.2.3; http://www.r-project.org/).

## 3 Results

### 3.1 Descriptive statistics

Overall, a total of 174 (54.5%) patients underwent neobladder *vs.* 145 (45.5%) ileal conduit urinary diversion (**Table 1**). Median follow-up was 43 months in ONB patients (interquartile range (IQR): 19–81) and 54 months in IC patients (IQR: 17–60) (p = 0.2). The median age was 68 (IQR: 61–74) and was significantly lower among individuals with neobladder (p < 0.001). Male individuals represented the majority of the study population (n = 271; 85.0%) and almost exclusively represented the neobladder strata (91.4%).

**Table 1:** Descriptive statistics for 319 patients with clinical bladder cancer (BCa) treated with radical cystectomy (RC) and neobladder or ileal conduit urinary diversion interviewed between January 2010 and December 2013.

Parameter	Overall	Neobladder (n=174, 54.5%)	Ileal conduit (n=145, 45.5%)	p-value
**Age** IQR	68 61-74	66 57-71	72 66-77	< 0.001
**QoL scale** IQR	67 50-83	67 50-83	67 50-83	0.4
**Gender** Male Female	271 (85.0) 48 (15.0)	159 (91.4) 15 (8.6)	112 (77.2) 33 (22.8)	< 0.001
**Chronic cardiac failure** No Mild Severe	174 (54.5) 130 (40.8) 15 (4.7)	80 (46.0) 88 (50.6) 6 (3.4)	94 (64.8) 42 (29.0) 9 (6.2)	< 0.001
**Peripheral vascular disease** (yes)	48 (15)	26 (14.9)	22 (15.2)	0.9
**Chronic kidney *disease*** (yes)	119 (37.3)	81 (46.6)	38 (26.2)	< 0.01
**Hypertension** No Mild Moderate Severe	115 (36.1) 160 (50.2) 42 (13.2) 2 (0.6)	60 (34.5) 98 (56.3) 15 (8.6) 1 (0.6)	55 (37.9) 62 (42.8) 27 (18.6) 1 (0.7)	0.03
**Neurological disease** (yes)	9 (2.8)	4 (2.3)	5 (3.4)	0.8
**Diabetes** (yes)	56 (17.6)	22 (12.6)	34 (23.4)	0.02
**Chronic arthritis** (yes)	26 (8.4)	8 (4.6)	18 (12.4)	0.02

QoL, quality of life; IQR, interquartile range.

Patient distribution across neobladder *vs.* ileal conduit groups did not significantly differ across the five centers that participated in the study (p = 0.4). However, the two groups significantly differed with regard to BMI and comorbidity profiles. Specifically, the median BMI was significantly higher among individuals with ONB *vs.* IC (27 *vs.* 26; p = 0.02). Several comorbidity profiles were significantly different between the two strata. Specifically, patients free from chronic cardiac failure (46.0% *vs.* 64.8%; p < 0.001), chronic kidney disease (53.4% *vs.* 73.8%; p < 0.01), and hypertension (34.5% *vs.* 37.9%, p < 0.03) were less represented in the ONB *vs.* IC strata. Conversely, patients free from diabetic disease (87.4% *vs.* 76.6%; p = 0.02) and chronic arthritis (95.4% *vs.* 87.6%; p = 0.02) were more represented in the neobladder compared to the ileal conduit strata. No difference was observed in the prevalence of peripheral vascular disease-free individuals (85.1% *vs.* 84.8%; p = 0.9) across the two groups.

### 3.2 Multivariable analysis

**Figure 1:**
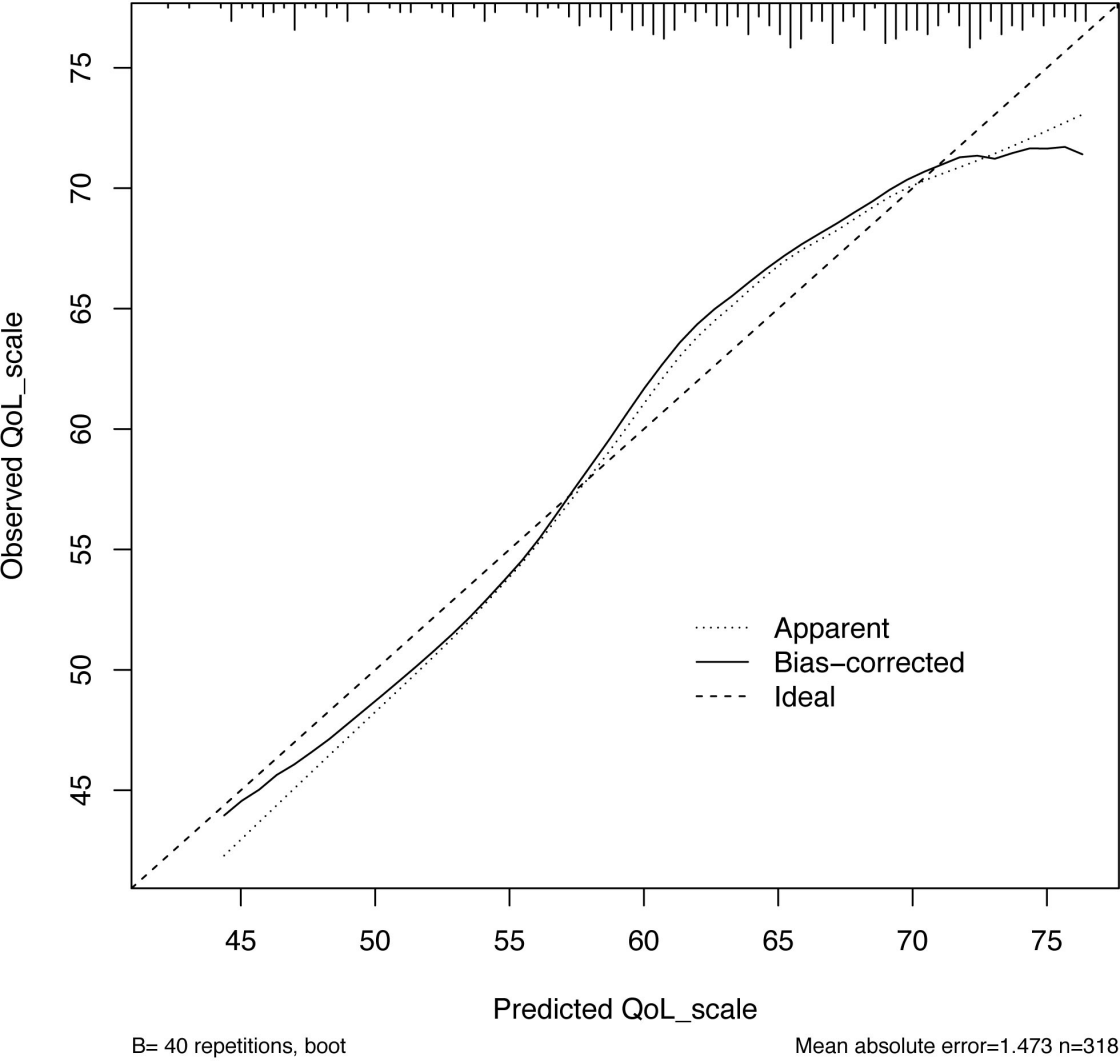
Calibration plot of observed scores versus predicted score of global health status/QoL score of the novel nomogram. QoL, quality of life.

**Figure 2:**
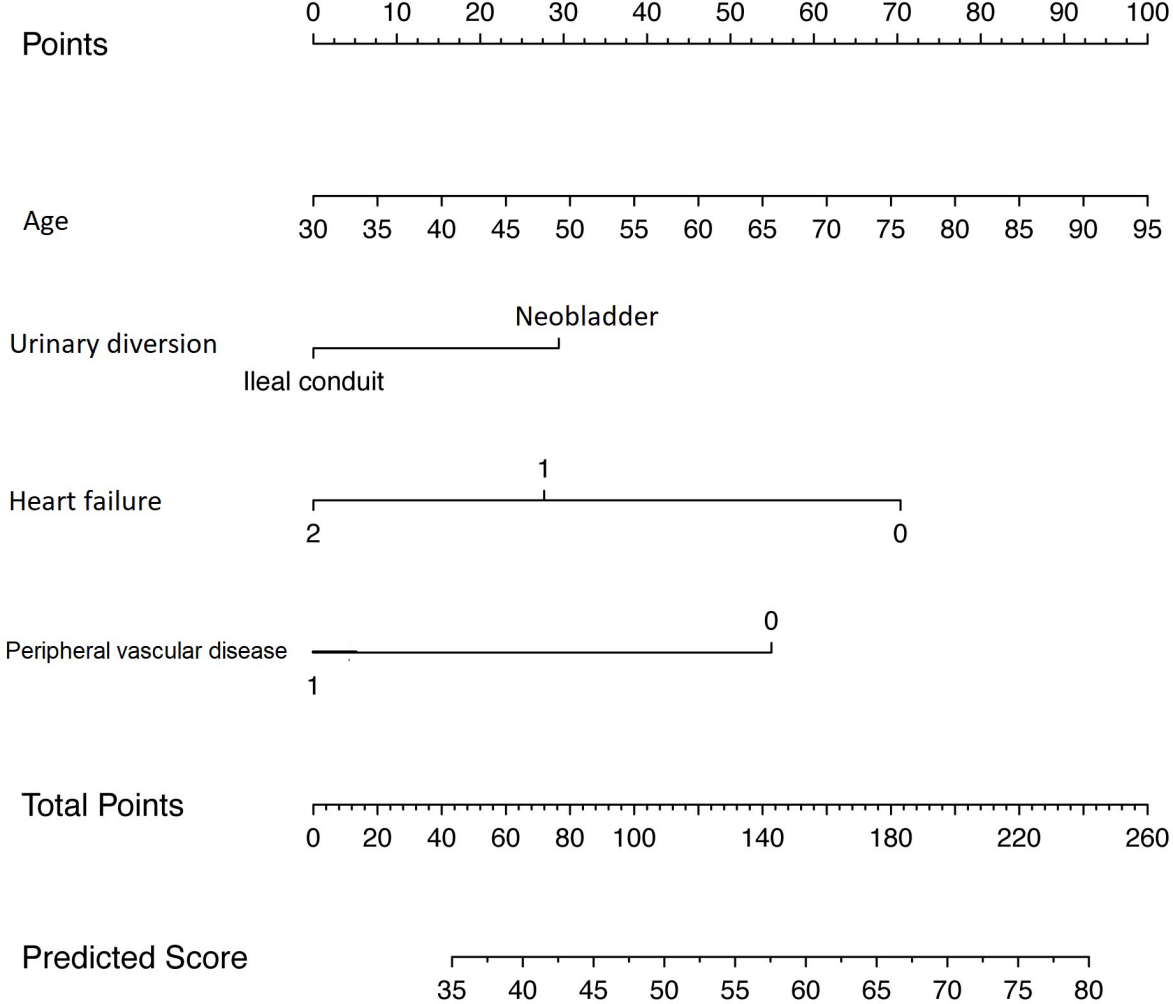
Novel nomogram predicting the global health/QoL score for patients diagnosed with BCa and treated with RC and neobladder or ileal conduit urinary diversion. Instructions: locate the patient’s age on the corresponding axis. Draw a line straight upward to the point axis to determine how many points toward the score of global health status/QoL the patient receives for his age. Repeat the process for each additional variable. Sum the points for each of the predictors. Locate the final sum on the total point axis. Draw a line straight down to find the patient’s predicted score on the global health status/QoL scale. QoL, quality of life; BCa, bladder cancer; RC, radical cystectomy.

Starting from a set of variables that could potentially have been included in the nomogram, we proceeded by testing several prediction models until a multivariable linear regression model predicting the global QoL score was developed using the most significant predictors as identified in the present study cohort (**Table 2**). Specifically, patient age, UD, chronic cardiac disease, and peripheral vascular disease emerged as the only statistically significant predictors for our criterion (all p < 0.03). The calibration plot of the developed prediction model showed a systematic overestimation of the predicted QoL score over the observed QoL scores, with a slight underestimation of observed QoL scores between 57 and 72 (**Figure 1**). The root mean square error (RMSE) of the developed prediction model was 23.6. RMSE is a way of representing the average distance of a data point from the fitted model. In other words, it is an index that quantifies how much a certain value, predicted with a model for a given observation, is close to the true value of said observation. RMSE, more specifically, is the square root of the mean squared error and is used to represent the average distance of a certain value from the fitted model. As a general rule of thumb, lower RMSE values indicate a better fit and a better prediction model. After leave-one-out cross-validation was performed, RMSE emerged as 24.0. The nomogram developed is shown in **Figure 2**.

**Table 2:** Multivariable linear regression analyses assessing the prediction of global health status/QoL among patients diagnosed with BCa and treated with RC and neobladder or ileal conduit urinary diversion.

Parameter	Slope	95% CI	p-Value
**Constant**	43.1	21.5–64.8	<0.001
**Age**	0.35/year	0.05–0.64	0.02
**Urinary diversion** (neobladder vs. ileal conduit)	6.67	0.92–12.4	0.02
**Chronic cardiac failure** Mild vs. no Severe vs. no	−9.68 −15.96	−15.48 to −3.88 −28.8 to −3.14	0.001 0.01
**Peripheral vascular disease** (yes vs. no)	−1.45	−20.1 to −4.76	<0.01

QoL, quality of life; BCa, bladder cancer; RC, radical cystectomy.

## 4 Discussion

RC followed by UD generally has a substantial impact on patients’ QoL (17). The possibility to predict preoperatively how the treatment could impact patients’ QoL could be important to fully inform the patients about the benefits and potential risks of available treatment options directing the decision-making process (2). However, QoL is a multi-dimensional reality, and consequently, the prediction of QoL outcomes is challenging (18, 19). The prediction of outcomes relevant to disease management has been generally based on physician judgment and risk classification systems (20, 21). These methodological strategies are not usable for multiple concomitant relevant factors that potentially lead to inaccurate predictions (22). In this regard, nomograms are alternative prediction tools based on statistical models that can incorporate multiple variables generating individualized predictive factors for each patient potentially improving clinical predictivity and thus evidence-based medical decision-making (5). As specific clinical predictivity of post-cystectomy QoL has been scarcely investigated, the type of UD was tested as a possible predictor of better overall post-cystectomy QoL and resulted in ONB significantly predicting better QoL at 3 months, but not at 12 months after RC compared to IC diversion (3). In a different study, the QoL measured at baseline and before RC was found to be an independent predictor of QoL score at 1 year post-cystectomy, suggesting that patients in whom QoL is considered satisfactory before the surgery could potentially have available psychosocial resources, allowing them to preserve QoL after RC (4). Moreover, in the same study, the female gender is associated with higher QoL scores, and the authors assumed that this could be associated with gender differences concerning coping mechanisms, family support, expectations, and personal values (4, 23).

In another study, the investigation of QoL’s prediction after RC was exclusively dedicated to patients with ONB (24). After having tested the potential predictive efficacy of multiple preoperative, intraoperative, and postoperative variables, only preoperative performance status, surgeon experience, and daytime incontinence resulted as independent predictors of good postoperative QoL outcomes. However, considering that globally most patients receive an IC urinary diversion, their findings can be applied only to a small portion of patients undergoing RC (25). Moreover, the authors did not assess the performance of their multivariable prediction model, and no validation was performed.

Thanks to our study, it was possible to develop the first nomogram for patients with BC undergoing RC to predict a post-cystectomy QoL outcome. This tool is capable to calculate an estimate of post-cystectomy global QoL outcome tailored for the individual patient based completely on known preoperative factors. This information can be useful for patients’ preoperative counseling considering the high importance of fully informing the patient about the benefits and side effects of treatment options, allowing them to actively participate in the decision-making process concerning the treatment. Moreover, the preoperative global QoL estimate may support the case management process, allowing to engage the proper healthcare network according to patient characteristics and comorbidities.

To achieve this goal, we tested the ability of patient characteristics and UD, specifically ONB or IC, to independently predict the EORTC QLQ-C30 global QoL outcome postoperatively. We chose the global health status/QoL as the endpoint of the model to allow a better comparison with the existing literature (26, 27). Indeed, we developed and internally validated a multivariable prediction model that estimates the post-cystectomy global QoL for the individual patient. Specifically, patient age, UD, chronic cardiac disease, and peripheral vascular disease emerged as the only statistically significant predictors for our criterion. As regards patient age, we found that higher median age at surgery was associated with a higher global QoL score post-cystectomy. This finding is in line with previous publications that reported higher QoL outcomes in elderly patients compared with younger ones (26). It is reasonable to assume that since younger patients may have higher expectations and more active lifestyles, radical surgery with its detrimental consequences on physical, social, and psychological dimensions could likely result in higher dissatisfaction levels (28, 29). Furthermore, in our multivariable prediction model, the type of UD emerged as a statistically significant predictor of global QoL score, and specifically, ONB was associated with higher levels of global QoL score when compared to IC urinary diversion. This result could be consistent with other contributions in the literature reporting better QoL outcomes for ONB compared to IC (5, 6). Indeed, when no contraindications exist, ONB may represent the most valuable option.

Comorbidities are generally associated with worst postoperative outcomes after RC. In our prediction model, chronic cardiac failure and peripheral vascular disease resulted in significant predictors of post-cystectomy global QoL score. Specifically, mild to severe heart failure and the presence of a peripheral vascular disease predict a decrease in the estimated global QoL score.

We tested the performance of the multivariable model by assessing the calibration, and we observed a systematic overestimation of the predicted global QoL score over the observed global QoL score with a modest underestimation for observed global QoL scores between 57 and 72. Moreover, the RMSE was 24.0 after performing leave-one-out cross-validation, suggesting adequate predictive accuracy.

Nevertheless, our study is not devoid of limitations. First, regarding socio-demographic data, the study considered age and gender. Other data such as marital status, education level, socioeconomic level, and area of residence were not considered in the statistical analyses. This is a limitation of the study, considering that these conditions could affect patients’ QoL. The nomogram was developed starting from a multivariable linear regression model that included patient age at surgery, UD, chronic cardiac disease, and peripheral vascular disease, based on the fact they represented the most significant predictors for QoL in our base model, and they can be evaluated preoperatively. However, other parameters that can impact lately on the patients’ QoL perception, such as BC pathological stages, postoperative complications, and daytime and nighttime continence (30, 31), have not been included in the study, representing a limitation for the predictive accuracy of the nomogram. Another limitation is due to the fact that women are under-represented in the sample, and therefore, global QoL prediction could be less accurate for them. Moreover, the nomogram needs to be externally validated to confirm the validity of our model in other settings by applying it to a contemporary sample of patients. Finally, our study is limited in relation to its retrospective modality and thus potentially influenced by patient selection biases typical of all retrospective series.

We suggest that future investigations perform an extensive external validation of the nomogram as a part of a prospective study in order to verify the predictivity of the model itself over time.

## 5 Conclusions

We developed a novel nomogram based completely on known preoperative factors for patients with MIBC undergoing RC to predict a mid-term QoL outcome that can support patients’ counseling.

## Data availability statement

The original contributions presented in the study are included in the article/supplementary material. Further inquiries can be directed to the corresponding author.

## Ethics statement

The study was approved by the Ethics Committees of Verona and Rovigo (protocol number 20097339KC). The patients/participants provided their written informed consent to participate in this study.

## Author contributions

SS was responsible for the conception and design of the work. AZ, EZ, AP, RC, FM, CL and FR were responsible for data acquisition and interpretation and for drafting the work. RT was responsible for data analysis.All authors contributed to the article and approved the submitted version.
